# Plant sex influences on riparian communities and ecosystems

**DOI:** 10.1002/ece3.10308

**Published:** 2023-07-12

**Authors:** River P. Scheuerell, Carri J. LeRoy

**Affiliations:** ^1^ Environmental Studies Program The Evergreen State College Olympia Washington USA

**Keywords:** dioecious, ecosystem function, herbivores, leaf chemistry, morphology, riparian zones, rivers, *salix*, streams, physiology, *populus*

## Abstract

Over the past several decades, we have increased our understanding of the influences of plant genetics on associated communities and ecosystem functions. These influences have been shown at both broad spatial scales and across many plant families, creating an active subdiscipline of ecology research focused on genes‐to‐ecosystems connections. One complex aspect of plant genetics is the distinction between males and females in dioecious plants. The genetic determinants of plant sex are poorly understood for most plants, but the influences of plant sex on morphological, physiological, and chemical plant traits are well‐studied. We argue that these plant traits, controlled by plant sex, may have wide‐reaching influences on both terrestrial and aquatic communities and ecosystem processes, particularly for riparian plants. Here we systematically review the influences of plant sex on plant traits, influences of plant traits on terrestrial community members, and how interactions between plant traits and terrestrial community members can influence terrestrial ecosystem functions in riparian forests. We then extend these influences into adjacent aquatic ecosystem functions and aquatic communities to explore how plant sex might influence linked terrestrial‐aquatic systems as well as the physical structure of riparian systems. This review highlights data gaps in empirical studies exploring the direct influences of plant sex on communities and ecosystems but draws inference from community and ecosystem genetics. Overall, this review highlights how variation by plant sex has implications for climate change adaptations in riparian habitats, the evolution and range shifts of riparian species and the methods used for conserving and restoring riparian systems.

## Graphical abstract

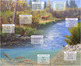

## 1 INTRODUCTION

### 1.1 What is dioecy?

Dioecious plants are species that form male and female reproductive organs on separate individuals, in contrast to monoecious plants that produce male and female flowers on the same plant or in “perfect” flowers that contain both male and female reproductive organs and are technically bisexual (Bawa, 1980a). The definition of dioecy can be either limited to strictly dioecious plants (as above) or be more expansive to include sub‐dioecious types: (1) polygamodioecious (or trioecious), (2) gynodioecious, and (3) androdioecious species (Bawa, 1980a; Cronk, 2022). For simplicity, in this review paper, we will limit our discussion to strictly dioecious plants with separate male and female individual plants. Sex determination in plants is variable and much more complex than the XY chromosome system in humans (Renner, 2014). Genetic controls of sex are common, but there may also be strong environmental controls in natural populations that influence sex expression and sex ratios on the landscape (Bawa, 1980b). For most of the plant species discussed in this review paper, the mechanism of sex determination is still unknown.

### 1.2 How common is dioecy?

Dioecy is phylogenetically widespread, appearing in 37 of 51 plant orders (Bawa, 1980a; Cronk, 2022), but at the same time, dioecy is relatively rare, found in only 7% of flowering plant genera and accounting for only 5–6% of angiosperm species (Renner, 2014; Renner & Ricklefs, 1995). Perhaps due to its rarity, it has been argued that dioecy is not a particularly successful mode of plant reproduction (Bawa, 1980a). However, dioecy has independently evolved from hermaphroditism an estimated 871 to 5000 times in angiosperms (Tonnabel et al., 2017). Whether the switch from hermaphroditism to dioecy occurs is likely influenced by pollinators, as hermaphrodites are more likely to rely on specialized pollinators, while dioecious species tend to rely on generalist pollinators or abiotic pollen dispersal (Bawa, 1980a; Delph, 1990; but see Renner & Feil, 1993). In some cases, dioecy has evolved without close plant–pollinator interactions, as for wind‐pollinated dioecious species like poplars, willows, and buckwheats (Pickup & Barrett, 2012). Dioecy is relatively more common in plants growing along streams and rivers, with over 30% of riparian plants in the Intermountain West and California (USA) being dioecious, including, notably, willows, cottonwoods, and box elders, key riparian tree species (Hultine et al., 2007b). The increased frequency of dioecious species in riparian zones may mean that dioecious plants play a particularly important role in riparian ecosystems.

### 1.3 How are male and female plants distributed?

The distributions of male and female individuals across the landscape can vary. Sex ratios of dioecious plants can range from female‐dominated (3:1 female: male), to equal (1:1), or male‐dominated (1:3 female: male) and can also differ significantly across habitat types (Munné‐Bosch, 2015). Two studies found 49.2% (Barrett et al., 2010) and 33% (Field et al., 2013) of dioecious species have balanced sex ratios. Of the remaining species with biased sex ratios, male bias occurs nearly twice as often (Field et al., 2013). The prevalence of a sex ratio bias in a particular direction varies phylogenetically, with male bias more common in vascular plants (Doust & Laporte, 1991; Dupont & Kato, 1999; Obeso, 2002). Sex ratios can also vary with pollination strategy, with plants relying on biotic pollination being male‐biased, whereas plants relying on abiotic pollination are more often female‐biased (Field et al., 2013). On a localized scale, sex ratios can vary with topography, resource availability (Munné‐Bosch, 2015), or be influenced by differential germination success or mortality, all of which can result in spatial segregation of the sexes.

### 1.4 Aims and questions: How might plant sex influence riparian systems?

**FIGURE 1:**
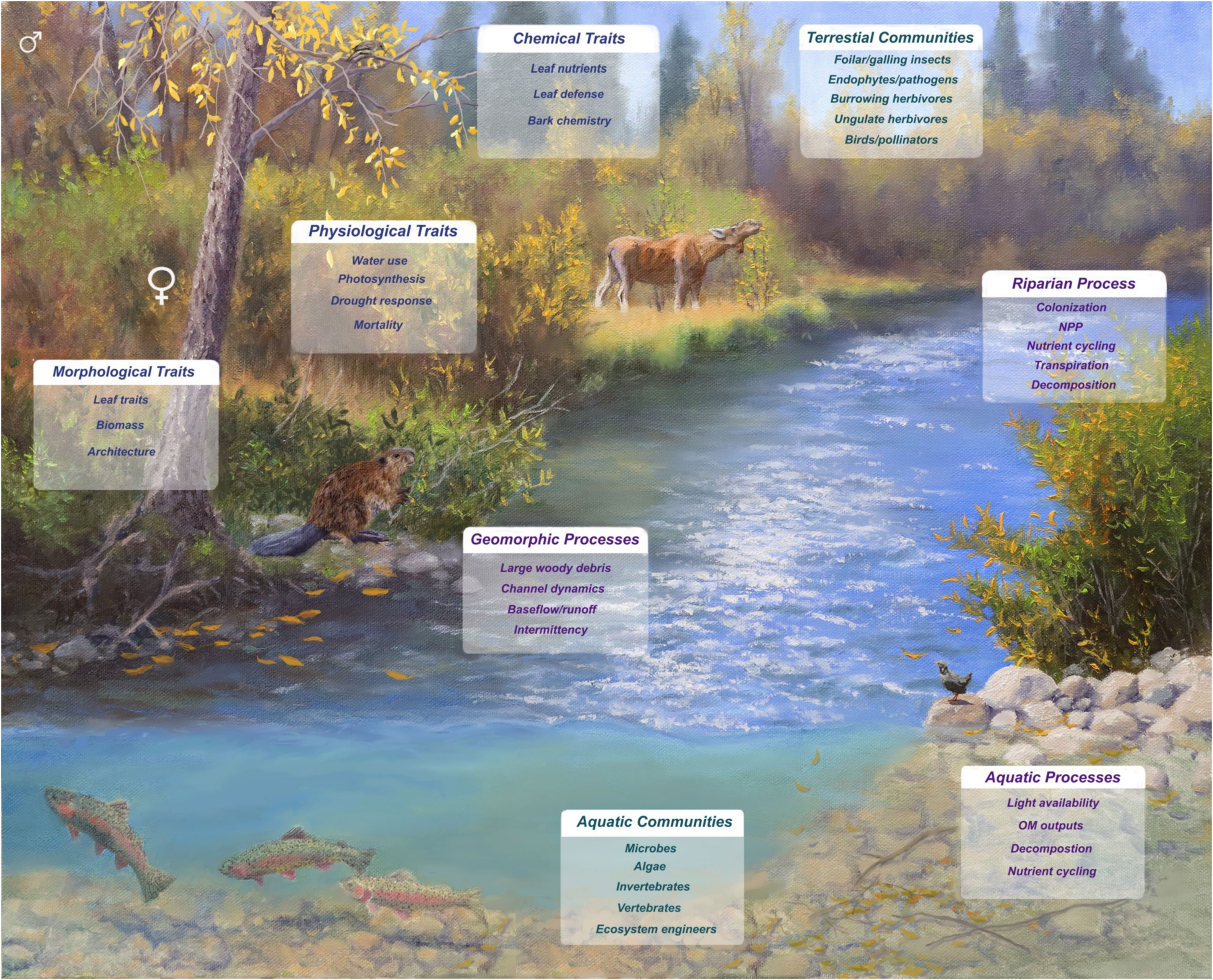
Potential influences of plant sex on riparian communities and ecosystem functions, from left in a spiral: morphological traits, physiological traits, chemical traits, terrestrial communities, riparian processes, aquatic processes, aquatic communities, and geomorphic processes.

This review paper will explore the influences of plant sex at several scales. First, we will discuss the influence of plant sex on morphological, physiological, and chemical plant traits at the plant level. Next, we will describe how these plant traits, controlled by plant sex, influence terrestrial community members and terrestrial ecosystem functions, specifically in riparian forests where dioecious species are common. Then, we will explore how plant traits and both riparian ecosystem processes and riparian community members can influence adjacent aquatic ecosystem functions and aquatic communities. Finally, we will explore how plant sex might, through these mechanisms, influence the physical structures of riparian systems (Figure 1) and their ability to adapt to impending climate changes and restoration actions (see Section 10).

## 2 METHODS

### 2.1 Eligibility criteria

Studies included in this systematic review explored the influences of plant sex on morphological traits, physiological traits, or chemical traits, for primarily riparian plant species. In addition, we included studies of plant sex on terrestrial community members, riparian ecosystem functions, aquatic community members, and the physical structure of riverine systems. We screened titles, abstracts, and full texts looking for studies examining plant sex. We collected information on both significant (direction and magnitudes of differences were collected) and non‐significant influences of plant sex at these various levels of ecological organization. When no studies of the influence of dioecy on these levels of ecological organization were available, we explored other plant genetic factors and within‐species genetic variation.

### 2.2 Selecting studies and collecting data

We used a comprehensive search method between September 2020 and September 2022 to find published studies across all journals and years of publication (1980–2021, with a few papers added from 2022 following review). We used Google Scholar and key search terms for each section of the paper along with the following terms: Dioecious OR Dioecy OR Plant sex. At least one author read each paper and if either significant or non‐significant influences of plant sex were found, the information was collected along with the bibliographic information (in Zotero). We collected data from text and figures directly, determining the direction and magnitude of any differences between males and females as well as the units of measurement for each reported difference.

### 2.3 Results of study selection process

We reviewed just over 200 papers and include data from 121 papers in this review. Papers that were not included in the review either did not directly address plant sex, did not study riparian plants, or the effects of plant sex were confounded with other treatments and difficult to disentangle. We did not register this review prior to analysis. We completed the PRISMA (Preferred Reporting Items for Systematic Reviews and Meta‐Analyses) checklist and included all relevant information.

## 3 MORPHOLOGICAL TRAITS

### 3.1 Leaf characteristics

There is some evidence that leaf characteristics in dioecious plants can differ between the sexes, and these differences can inform our understanding of broader‐scale resource allocation strategies. According to a large meta‐analysis, male plants tend to exhibit larger leaves (Cornelissen & Stiling, 2005). Leaf traits can also vary by sex with environmental conditions and resource availability. For example, for *Populus cathayana* (Manchurian poplar, Salicaceae), when water was plentiful, leaf characteristics did not significantly differ by plant sex, whereas under drought conditions, females exhibited relatively lower specific leaf areas (Xu et al., 2008). Variation in leaf morphological traits may lead to differences in overall biomass allocation and/or interactions between plants and their environments.

### 3.2 Aboveground and belowground allocation

Intersexual differences in aboveground and belowground allocation and the plasticity of this allocation may play a role in understanding responses to stressors like drought and flooding. For example, in *Populus cathyana* (Manchurian poplar, Salicaceae) differences in root growth have been observed in response to the sex of the roots of nearby plants (Dong et al., 2017), and females exhibited comparative increases in root dry mass in response to inoculation with arbuscular mycorrhizal fungi (AMF; Wen‐Tong et al., 2019). Since root growth in riparian plants can influence erosion and stream channel dynamics, this could be one mechanism by which plant sex could influence the physical structure of riparian systems (see Section 9). In *Populus cathyana*, water stress is largely influenced by root processes (Han et al., 2013), and so drought stress may be greater for female plants. In response to flooding, the ability to shift allocation is important, and male *Populus cathyana* tend to suffer lower mortality under complete submergence (Su et al., 2016).

### 3.3 Plant architecture

Given intersexual differences in resource allocation and morphological leaf traits, it is probable that overall plant architecture may also vary with plant sex. In *Salix suchowensis* (shrub willow, Salicaceae), females exhibited greater dry weight, height, and greater ground diameter (Yang et al., 2020). Patterns vary for *Populus*, with male *Populus cathayana* (Manchurian poplar, Salicaceae) displaying greater height growth and basal diameter relative to females in one study (Xu et al., 2008), but female *P. cathayana* displaying greater above‐ and belowground biomass in another study (Chen et al., 2021). Although not yet examined by plant sex, there is evidence for a genetic basis to plant fractal geometry in the dioecious *Populus angustifolia*, *P. fremontii*, and the *P. angustifolia* × *P. fremontii* hybrid system, with implications for community structure and complexity (Bailey, Bangert, et al., 2004). Regardless, it is clear that differences in plant size and structure may influence plant function and physiology.

## 4 PHYSIOLOGICAL TRAITS

### 4.1 Plant water use and transpiration

It has been suggested that greater reproductive costs for female plants may drive greater water consumption in female trees (Braatne et al., 2007; Darwin, 1877; Dawson et al., 1990; Gehring & Linhart, 1993; Gehring & Monson, 1994; Hultine et al., 2007b; Putwain & Harper, 1972). Over 50 years of research in a variety of species has documented sex‐based differences in plant transpiration, conductance, or water use efficiency (WUE). In multiple species, female individuals have a tendency toward lower stomatal regulation (higher stomatal conductance; *g*_s_) and lower WUE, resulting in higher transpiration (*E*) rates. For example, female *Ilex aquifolium* (common holly, Aquifoliaceae) exhibited higher water stress (δ^13^C, the stable isotope of C that can exhibit water stress) than males under xeric conditions (Retuerto et al., 2000), suggesting lower WUE. In *Acer negundo* (boxelder, Sapindaceae), females also exhibited greater stomatal conductance, greater leaf‐level resource uptake capacity, and lower water use efficiency (Hultine et al., 2007a). Similarly, in *Salix arctica* (arctic willow, Salicaceae), females exhibited greater stomatal conductance (*g*_s_) in wet habitats when soil and root temperatures were below 4°C and possibly greater WUE as indicated by less negative δ^13^C (Dawson & Bliss, 1989). In xeric habitats, male *S. arctica* maintained higher stomatal conductance compared with females (Dawson & Bliss, 1989). Both contrasting and mixed results were shown for *Salix polaris* (polar willow, Salicaceae) in terms of stomatal resistance, depending on a variety of environmental conditions (Crawford & Balfour, 1983). The consensus is that male woody plants tend to have a more conservative water use strategy, which may have ecohydrological implications. Adaptations that allow plants to maintain transpiration rates through extreme conditions may lead to increased drought tolerance and have evolutionary consequences in the face of climate changes (see Section 10).

### 4.2 Drought resistance

Drought is recognized as a crucial limiting factor for both plant growth and ecosystem productivity (Passioura, 1996) and there is a body of evidence showing poorer drought tolerance in female plants compared with males, especially for members of the Salicaceae. Under drought conditions, male *Populus cathayana* (Manchurian poplar, Salicaceae) displayed greater leaf area, woody biomass, and dry matter accumulation relative to females (Xu et al., 2008). These traits were linked specifically with increased male root growth and productivity patterns. For example, in grafted *P. cathayana*, male roots resulted in reduced limitations on growth and gas exchange during drought regardless of the sex of the shoots (Han et al., 2013). In addition, in *Salix arctica* (arctic willow, Salicaceae), males exhibited greater tissue turgor pressure at more depleted tissue water levels relative to females, due to better osmotic adjustment capabilities (Dawson & Bliss, 1989). Greater female susceptibility to drought could help explain female‐biased sex ratios close to rivers and streams (Hultine et al., 2007a, 2007b; LeRoy, Ramstack Hobbs, et al., 2020), where surface water availability is more stable and groundwater is closer to the surface.

### 4.3 Plant mortality

The prevailing pattern in plant mortality is of higher female mortality rates under stressful conditions (Ågren, 1988; Ataroff & Schwarzkopf, 1992; Barrett et al., 2010). This is observed in *Oemleria cerasiformis* (osoberry, Rosaceae), for which 59% of dead and dying genets were female (Allen & Antos, 1993). Similarly, in *Baccharis dracunculifolia* (rosemary‐of‐the‐field, Asteraceae), shoot mortality in females was 38.4% while in males it was 23.1% (Espírito‐Santo et al., 2003). It is thought that sex‐differential mortality rates may have to do with slower growth rates for female plants (Allen & Antos, 1993) but may also be due to differences in reproductive effort (but see Midgley, 2022). In *O. cerasiformis*, the only observed sex‐linked difference was in floral structure, and male‐biased sex ratios were present only at post‐reproductive maturity, pointing to reproductive effort as a potential driver of observed female‐biased mortality (Allen & Antos, 1988). Relatedly, in *Rubus chamaemorus* (cloudberry, Rosaceae), mortality of fruit‐bearing female ramets was greater than that of male ramets, but only at non‐shaded sites (Ågren, 1988), pointing to potential environmental interactions with sex‐based mortality. In *Salix sachalinensis* (Japanese fantail willow, Salicaceae), no mortality differences across sexes were found at any stage, including during reproduction (Ueno et al., 2007; Ueno & Seiwa, 2003). Contrary to the norm, willow populations are routinely female‐biased (Che‐Castaldo et al., 2015; Crawford & Balfour, 1983; Dawson & Bliss, 1989). The tendency for female willows to grow closer to water sources (Che‐Castaldo et al., 2015; LeRoy, Ramstack Hobbs, et al., 2020) may contribute to the lack of observed female‐biased mortality in several studies.

## 5 CHEMICAL TRAITS

### 5.1 Leaf nutrient content

In many dioecious plants, there are intersexual differences in nutrient content of leaves, bark, flowers, or fruits, with potentially wide‐reaching influences on riparian ecosystems. In several key tree species, overall nutritional content of females is greater. *Spondias purpurea* (purple mombin, Anacardiaceae) females were found to have greater nutritional quality than males (Maldonado‐López et al., 2014). In another study, where there was high P supply, *Populus cathayana* (Salicaceae) females had higher leaf P concentrations than males (Xia et al., 2020). In addition, leaf N concentrations were found to be greater in females of *Lithraea molleoides* (aroeira blanca, Anacardiaceae; Galfrascoli & Calviño, 2020). However, bark nitrogen concentrations were higher in male *Populus tremula* (Eurasian aspen, Salicaceae; Hjältén, 1992) as were nitrogen levels in needles of male *Juniperus communis* (common juniper, Cupressaceae; Rabska et al., 2020).

In dioecious shrubs in particular, there is often evidence of higher nutritional content in males. Male Sitka willows (*Salix sitchensis*, Salicaceae) exhibit greater leaf nitrogen concentrations (LeRoy, Ramstack Hobbs, et al., 2020; Ramstack Hobbs et al., 2022), and male *Salix caprea* (goat willow, Salicaceae) have higher bark nitrogen concentrations (Hjältén, 1992). Male flowers of *Rubus chamaemorus* (cloudberry, Rosaceae) had greater overall nutrient content (Ågren, 1988). However, some shrub species exhibit no intersexual differences in nutritional content or quality. Leaf nitrogen did not differ between the sexes for *Baccharis halimifolia* (groundsel tree, Asteraceae; Krischik & Denno, 1990), *Lindera benzoin* (spicebush, Lauraceae; Cipollini & Whigham, 1994), *Oemleria cerasiformis* (osoberry, Rosaceae; Allen & Antos, 1988), or *Myrica gale* (sweet gale, Myricaceae; Mizuki et al., 2018).

Where there are intersexual differences in nutritional quality of plants, there can be significant feedbacks regarding herbivores, insects, and even insect predators, and the chemical makeup of leaves can influence decomposition rates (see Sections 6 and 7, respectively). For instance, higher C:N ratios in female willow leaves that make them less nutritious for herbivores and also lead to slower in‐stream leaf litter decomposition patterns (LeRoy, Ramstack Hobbs, et al., 2020). This influence is compounded by increasingly female‐biased sex ratios close to stream banks (LeRoy, Ramstack Hobbs, et al., 2020), illuminating how patterns of spatial segregation of the sexes may interact with differences in nutrient content to influence riparian ecosystems.

### 5.2 Leaf defense compounds

It is generally thought that female plants allocate more resources to defense compound production than males, in part due to their slower growth rates and propensity to grow in resource‐rich areas (Pickering & Arthur, 2003), but a newer meta‐analysis calls this generalization into question (Sargent & McKeough, 2022). Condensed tannins are an important defense compound with wide‐reaching effects on terrestrial and aquatic ecosystems, including nitrogen mineralization, mycorrhizal interactions, herbivory, and microbial activity, which can lead to decreased leaf litter decomposition rates (Schweitzer, Madritch, et al., 2008). Females of some willow species have been found to contain higher tannin concentrations (Elmqvist et al., 1991; but see Nissinen et al., 2018, which shows no sex‐based differences). Higher phenolic compound concentrations have been found in female *Clusia fluminensis* (dwarf pitch apple, Clusiaceae; Guimarães et al., 2021) as well as in female *Juniperus communis* (common juniper, Cupressaceae; Rabska et al., 2020). If a connection between plant sex and tannins could be made, it could help us understand linkages between plant sex and beavers, key riparian ecosystem engineers, who have been shown to avoid cottonwoods with high bark tannins (Bailey, Schweitzer, et al., 2004). In addition to higher tannin concentrations, female willows have been found to contain higher salicortin, chlorogenic acid, and phenolic compounds (Hjältén, 1992; Nissinen et al., 2018). However, in *Populus tremula* (European aspen, Salicaceae), greater phenolic compound content was found in males (Hjältén, 1992). In contrast to patterns shown for woody members of the family Salicaceae, no intersexual differences in tannin concentrations were found for *Baccharis dracunculifolia* (green propolis, Asteraceae; Espírito‐Santo et al., 2003), though *B. dracunculifolia* may be an outlier as it has been shown to display no intersexual differences in several traits where differences were found for other dioecious plants (described in previous sections).

## 6 TERRESTRIAL COMMUNITIES

### 6.1 Foliar insects/insect predators

There is a substantial body of evidence to suggest that plant sex can affect host suitability for foliar insects, and in turn, drive higher trophic‐level interactions with insect predators (Romero‐Pérez et al., 2020). Intersexual differences in host plant quality might be driven by differences in nutritional suitability (Maldonado‐López et al., 2014), nectar production (Dötterl et al., 2014; Petry, 2016), or pollen production (Ågren et al., 2012; Cole & Ashman, 2005). In both female *Silene acaulis* (cushion pink, Caryophyllaceae) and *Valeriana edulis* (tobacco root, Caprifoliaceae), female plants have been shown to support greater quantities of arthropods than their male counterparts (Lortie & Reid, 2012; Petry, 2016). In addition, female *V. edulis* plants tend to support more aphids and ants than male *V. edulis* plants (Petry, 2016). However, in *Baccharis salicifolia* (mulefat, Asteraceae), ants and their tending aphids were more prevalent on male plants (Abdala‐Roberts et al., 2016). Despite these individual examples, when assessed broadly through meta‐analysis, the effect of plant sex on insect herbivory is widely variable, leading to no overall significant difference between males and females, even though specific species can respond strongly to herbivores based on plant sex, with riparian plants showing both male and female preferences (Sargent & McKeough, 2022).

These disparities can then drive higher trophic‐level interactions (Romero‐Pérez et al., 2020). Driven by the greater density of aphids on female *V. edulis* plants, a greater density of aphid predators can be found on those plants (Petry, 2016). In the case of *Salix cinerea* (gray willow, Salicaceae), the herbivore, *Phratora vulgatissima* (blue willow beetle, Chrysomelidae, Coleoptera) is not inherently better suited to either sex host plant, but the herbivore's predator, *Anthocoris nemorum* (common flowerbug, Anthocoridae, Hemiptera), develops faster on male willows (Kabir et al., 2014). In contrast, the predator's host plant sex preference drives the herbivore to inhabit female *S. cinerea* plants in higher numbers (Kabir et al., 2014). The significant influence of plant sex on ants and aphids on *B. salicifolia* (mulefat, Asteraceae) did not result in a significant influence on parasitoid predators (Abdala‐Roberts et al., 2016).

Nutritional quality is not the only factor that can influence an insect's host preference. In a related willow, *Salix viminalis* (basket willow, Salicaceae), herbivores laid more eggs on female plants and survival of the predator was greater, but only under laboratory conditions (Moritz et al., 2017). For instance, two leaf beetles (Chrysomelidae) fed preferentially on male *Baccharis halimifolia* (groundsel tree, Asteraceae) leaves, for which there were no intersexual nutritional differences, but because the male leaves were more tender (Krischik & Denno, 1990).

### 6.2 Galling insects

Galling insects are ecosystem engineers with strong influences on non‐galling insect communities (Wimp et al., 2005) and ecosystem functions like leaf litter decomposition (LeRoy, Fischer, et al., 2020) that may be influenced by host plant sex. A meta‐analysis found that plant sex played a strong role in galling insect abundance, with differences in phenological, morphological, and nutritional traits given as possible explanations (Cornelissen & Stiling, 2005). In *Acer opalus* (Italian maple, Sapindaceae), male plants have been shown to be more heavily influenced by galling insects in both quantity and intensity (Verdú et al., 2004). Larger gall size and increased reproduction of *Slavum wertheimae* (a gall‐forming aphid, Pemphigidae, Homoptera) was found on male *Pistacia atlantica* (Persian turpentine, Anacardiaceae; Wool & Bogen, 1999), while only male *Clusia fluminensis* (dwarf pitch apple, Clusiaceae) were targeted by a fly galler (Cecidomyiidae, Diptera), likely due to higher phenolic compound production in females (Guimarães et al., 2021). However, there are departures from this trend. No intersexual difference in *Phyllocolpa leavitti* (sawfly, Tenthredinidae, Hymenoptera) density on *Salix discolor* (glaucous willow, Salicaceae) could be found, but survival rates of *P. leavitti* were twice as high on female host plants (Fritz et al., 2003). As can perhaps be expected given their general lack of notable intersexual differences in other interactions, *Baccharis* spp. (Asteraceae) have been found to have no sex‐linked disparities in galling insect abundance across various studies (Carneiro et al., 2006; Espírito‐Santo et al., 2012; Faria & Fernandes, 2001; Marques & Fernandes, 2016; Ribeiro‐Mendes et al., 2002). Galling insects can be responsible for chemical induction in plants (insect‐stimulated increases) of phenolic compounds and tannins in host plants (Hall et al., 2017). Chemical induction can be lasting and influence “afterlife” effects like decomposition rates (Findlay et al., 1996) and could link leaf chemistry and leaf herbivory to both terrestrial and aquatic ecosystem functions (see Section 7).

### 6.3 Microbial symbionts

Plants interact with a variety of microbial symbionts: endophytes, mycorrhizae, phyllosphere microbes, and pathogens; each of these groups can be influenced by the sex of their host plants. Endophytes can drive a diverse array of processes for host plants, with some evidence for differential effects on male and female plants. Endophytes of the genus *Epichloë* are known to reduce infection by pathogens, both in the symbiont plant and its seeds (Iannone et al., 2017; Li et al., 2007; Pérez et al., 2020; Vignale et al., 2013). In addition, they can increase defense compound production, increase drought tolerance and biomass of hosts (Clay, 1988; Clay & Schardl, 2002; Crawford et al., 2010; Malinowski & Belesky, 2000; Saikkonen et al., 2010; Schardl et al., 2007), and even influence decomposition rates (Lemons et al., 2005; Purahong & Hyde, 2011). While there is insufficient research on the interactions between *Epichloë* endophytes and plant sex to make many broad conclusions, there are a few notable results. In the dioecious grass *Poa bonariensis* (Poaceae), decreased incidence of infection by other endophytic fungi was influenced by the presence of *Epichloë* spp. and was more pronounced in males (McCargo et al., 2020). A study on the phyllosphere community of *Populus cathayana* (Manchurian poplar, Salicaceae) found significant intersexual differences in the quantities of Ascomycota and Basidiomycota fungi as well as Proteobacteria and Planctomycetes bacteria (Liu et al., 2021). Specifically, more fungi of the genera *Phoma* and *Aurobasidium* were found in males, and more fungi of the genera *Suillus*, *Venturia*, and *Elmeria* were found in females (Liu et al., 2021). More research on the influences of these observed intersexual differences in endophyte host plants would inform our understanding of the role of plant sex in other situations, including herbivory, decomposition, and net primary production. Given the propensity for endophytes to confer properties to the next generation through seeds, these interactions may also help explain evolutionary patterns, colonizer species efficacy, and observed sex ratios across landscapes.

Mycorrhizal fungi can positively influence plant performance, though these effects can differ by plant sex (Vega‐Frutis et al., 2015). Greater colonization of female plants by arbuscular mycorrhizae was found in 70% of tropical tree species (Vega‐Frutis et al., 2015). It is suggested that these intersexual differences in mycorrhizal colonization could be driven by differences in resource allocation, in light of the large resource demands of arbuscular mycorrhizae (Vega‐Frutis et al., 2015; Vega‐Frutis & Guevara, 2009). In *Populus cathayana* (Manchurian poplar, Salicaceae), arbuscular mycorrhizae formation benefitted male trees more, particularly in aiding drought tolerance (Li et al., 2020).

Limited research exists on pathogens and rusts in relation to plant sex. However, infection by the pathogen *Phratora vulgatissima* was found to more seriously influence females of *Salix viminalis* (basket willow, Salicaceae; Moritz et al., 2017). Additionally, the smut fungus *Micobrotryum lychnidis‐dioicae* has been shown to cause partial sex reversal in female *Silene latifolia* (white campion, Caryophyllaceae; Zemp et al., 2015). Genetic variation in cottonwoods (*Populus fremontii*, *Populus angustifolia*, and their hybrids, Salicaceae) has been linked to differences in leaf pathogen communities (Busby et al., 2013), but an exploration of plant sex is needed. Additionally, susceptibility to *Melampsora* spp. foliar rusts has been genetically linked in *Populus nigra* (black poplar, Salicaceae; Legionnet et al., 1999) and *Populus deltoides* (Eastern Cottonwood, Salicaceae; Thielges & Adams, 1975). Although few studies have explored plant sex directly, variation in rust susceptibility based on genetic variation may portend plant sex influences.

### 6.4 Mammalian herbivores

There are fewer studies on mammalian herbivores than insect herbivores with regard to plant sex, but some small mammals and ungulates have been shown to respond to the sex of plant forage in several studies. Research on hare herbivory exhibits a pattern where male plants are more targeted for herbivory across several species in the Salicaceae, showing that *Populus tremula* (Eurasian aspen), *Salix caprea* (goat willow), and to a lesser extent, *Salix pentandra* (bay willow), are all preferred by small mammalian herbivores (Hjältén, 1992). Higher nitrogen content in the bark of male *P. tremula* and *S. caprea* plants is offered as a potential driver of this outcome (Hjältén, 1992). Similarly, it has been suggested that in grazing‐limited areas, herbivory by bettongs and bilbies (small Australian marsupials) could lead to increased recruitment of female *Dodonaea viscosa* (hopbush, Sapindales; Munro et al., 2009). It has been noted that animal dispersal of seeds and fruits and male‐biased sex ratios are emblematic of tropical dioecious woody plants (Bawa, 1980b; Ibarra‐Manríquez & Oyama, 1992). These studies show that small mammals may help perpetuate either female‐ or male‐biased sex ratios through sex‐biased herbivory, but their overall influences may be complicated by broader ecological contexts and interactions with other organisms, such as large ungulate herbivores.

Ungulate herbivory is thought to have a significant influence on the vegetation of riparian ecosystems, with reduced tree and shrub heights along with reduced groundcover observed (Hood & Bayley, 2009). Introduction of ungulates can also affect the sex ratios of dioecious plants (Graff et al., 2013), but interestingly, we see a reversal in the trend identified above (where small mammal and burrower herbivory led to female‐biased sex ratios). In this example, where sheep were allowed to graze, *Poa ligularis* (meadow grass, Poaceae) sex ratios became more male‐biased, despite the female plants' greater defense compound production (Graff et al., 2013). A possible explanation is that defense compound production could be overshadowed by distance from relatively less desirable plants when it comes to mammalian herbivory (Atsatt & O'Dowd, 1976; Graff & Aguiar, 2011; Graff et al., 2013; Milchunas & Noy‐Meir, 2002). Given large differences in defensive compounds, growth strategies, and differential browsing by small mammals on male and female plants, the limited evidence relating to ungulate browsing may reflect a paucity of research on this important topic. This is accentuated by the influence of ungulates on riparian vegetation, which can affect nutrient cycling via herbivory‐induced greenfall (Maschinski, 2001) and alter overall riparian channel structure (Section 9).

### 6.5 Birds and other pollinators

Birds can interact with dioecious plants through insect predation, seed dispersal, and pollination. Given the influences of plant sex on insect communities outlined above, and the documented feedbacks for other insect predators, it stands to reason that insect predation by birds could also be influenced by plant sex. While this has not been studied directly, there is strong evidence for plant genetics and within‐species variation in plants influencing insect predation by birds (Bailey et al., 2006; Dickson & Whitham, 1996; Smith et al., 2011), with ramifications in terms of tree growth under herbivore release (Bridgeland et al., 2010). In addition to feeding on insects living on plants, some birds eat seeds/fruits from dioecious plants and zoochory often goes in concert with dioecy (Oliveira, 1996). There is a documented increased in seed dispersal of *Juniperus communis* seeds by birds of the genus *Turdus* linked to fruit rewards from female *Juniperus sabina* resulting in increased *J. communis* germination under female *J. sabina* nurse plants (Verdú & García‐Fayos, 2003). However, most riparian plants are wind‐pollinated and use either wind or water‐dispersal methods for seeds (Renner, 2014). Furthermore, wind pollination is predominant among dioecious plants (Renner & Ricklefs, 1995), and dioecious plants are thought to rely less on bird and bat pollination (Renner & Ricklefs, 1995), with only minor reliance on insect pollination (Renner & Feil, 1993), particularly in the tropics (Bawa, 1980b; Ibarra‐Manríquez & Oyama, 1992; Renner & Feil, 1993; Renner & Ricklefs, 1995). A lack of pollinator availability over long periods of time has been hypothesized to have influenced the evolution of dioecy in several species (Scobell & Schultz, 2005).

## 7 RIPARIAN ECOSYSTEM FUNCTIONS

### 7.1 Pioneer species efficacy

Differences in resource demands between the sexes can influence the efficacy of key riparian pioneers, which in turn, can drive or perpetuate spatial segregation of the sexes. For example, female plants often require more resources such as water and nitrogen, and thus are more likely to be found in closer proximity to streams (Che‐Castaldo et al., 2015; LeRoy, Ramstack Hobbs, et al., 2020). For wind‐pollinated species, spatial segregation of the sexes is moderated by the need for sufficient proximity for reproduction, and reproductive costs can also be high for males, due to large quantities of pollen production (Antos & Allen, 1990; Ashman & Baker, 1992; Chapin, 1989; Midgley, 2022). This pattern has been observed in *Acer negundo* (boxelder, Sapindaceae) where sex ratios within a meter of the riverbank were found to be female‐biased, while outside of that area, sex ratios were male‐biased (Hultine et al., 2007a).

Potentially also linked to resource demands, intersexual differences in mortality across differing microsites could be another driver of spatial segregation of the sexes (Bierzychudek & Eckhart, 1988). In *Lindera melissifolia* (pond berry, Lauraceae) it has been suggested that greater capabilities for interspecific competition by male individuals might make them better at establishing, leading to observed male‐biased sex ratios (Hawkins et al., 2009). However, the specific characteristics of riparian systems make this perhaps less applicable. Riparian disturbance zones are often the result of flooding, which in contrast to most disturbance zones, are resource‐rich and favor females. This matches patterns that have been observed for both vegetatively and sexually reproductive *Salix* spp. (willows, Salicaceae). Although clonal fragments of either sex are equally likely to propagate, female‐biased sex ratios are preserved in riparian zones due to a greater quantity of potential propagules from female plants stemming from the existing female bias in established riparian communities (Che‐Castaldo et al., 2015). *Salix* spp., along with other key dioecious riparian species, take woody or shrubby forms and rely on wind pollination with males dominating on upper terraces. Wind‐pollinated dioecious shrubs are often female‐biased (Field et al., 2013; Sinclair et al., 2012). Thus, interspecific differences in intersexual reproductive resource allocation should be considered, and such differences could guide a more nuanced understanding of observed sex ratios. We do not yet understand the mechanisms for biased sex ratios in most species, so more detailed studies of these mechanisms are needed.

### 7.2 Net primary production

The general trend is faster growth in male plants leading to greater net primary production (NPP), though there are some notable and important exceptions in riparian systems. In keeping with this, a large meta‐analysis across many plant families found that male plants produced more and larger leaves, longer stems, and grew faster overall (Cornelissen & Stiling, 2005). Faster growth rates in males were also found in *Populus yunnanensis* (Yunnan poplar, Salicaceae), with the lower growth rates of females exacerbated when exposed to copper and lead (Peng et al., 2020). Reproductive effort is a commonly cited reason for these differences, since some males produce fewer or smaller flowers, but meta‐analysis shows that both sexes allocated an equal proportion of aboveground biomass to reproductive structures overall (Cornelissen & Stiling, 2005). For *Auracaria araucana* (monkey puzzle, Araucariaceae), a masting species, reproductive effort‐influenced intersexual differences in net primary productivity plays out in cycles, with male growth reduced in high pollen production years and female growth reduced in the succeeding high seed production years (Hadad et al., 2021). It is well‐documented that, for some members of the Salicaceae—a key riparian group—the above patterns of increased male performance do not apply. For instance, female *Populus cathayana* (Manchurian poplar, Salicaceae) trees were found to have faster growth rates than males (Chen et al., 2021).

In some cases, there are also documented sex differences in growth responses to temperature. Increased temperature positively influenced short‐term peak season net C assimilation in female *Salix arctica* (arctic willow, Salicaceae), but negatively affected this measure in males (Jones et al., 1999). Perhaps relatedly, growth rates of female *Auracaria araucana* (monkey puzzle, Araucariaceae) were greater at warmer sites, while male growth rates were greater at colder sites (Rozas et al., 2019). A broader understanding of sex‐specific growth rates and NPP across variable environmental conditions would aid in understanding how climate changes may shape future populations and ecosystems (see Section 10).

### 7.3 Terrestrial nutrient cycling

While there has been limited direct research on the influence of plant sex on terrestrial nutrient cycling, strong influences of genetic variation on leaf chemistry traits and nutrient cycling may provide insights into the potential influences of plant sex. Condensed tannins (CT) in *Picea abies* (Norway spruce, Pinaceae) have been linked to reduced terrestrial nitrification rates and, in nitrogen‐poor soils, reduced carbon mineralization (Adamczyk et al., 2013). Condensed tannin concentrations in foliage among genotypes of *Populus* (cottonwood, Salicaceae) were found to be genetically influenced and accounted for 55–65% of the observed differences in N mineralization rates (Schweitzer et al., 2004). Genotypic variation in CT concentrations in *Populus* is also correlated with differences in microbial biomass and nutrient cycling (Schweitzer, Bailey, et al., 2008). There is some limited evidence that female plants produce more CT than males (Elmqvist et al., 1991; but see Nissinen et al., 2018; Sargent & McKeough, 2022, which show no sex‐based differences), pointing to a potential link between female plants and reduced nitrification rates, but more research on this is needed to establish a firmer connection. As for phosphorous cycling, in *Simarouba amara* (paradise tree, Simaroubaceae), females increased the extractable phosphorous levels in the soil around them, while males had no discernible effect on phosphorous (Rhoades et al., 1994).

### 7.4 Stand‐level water use and transpiration

**FIGURE 2:**
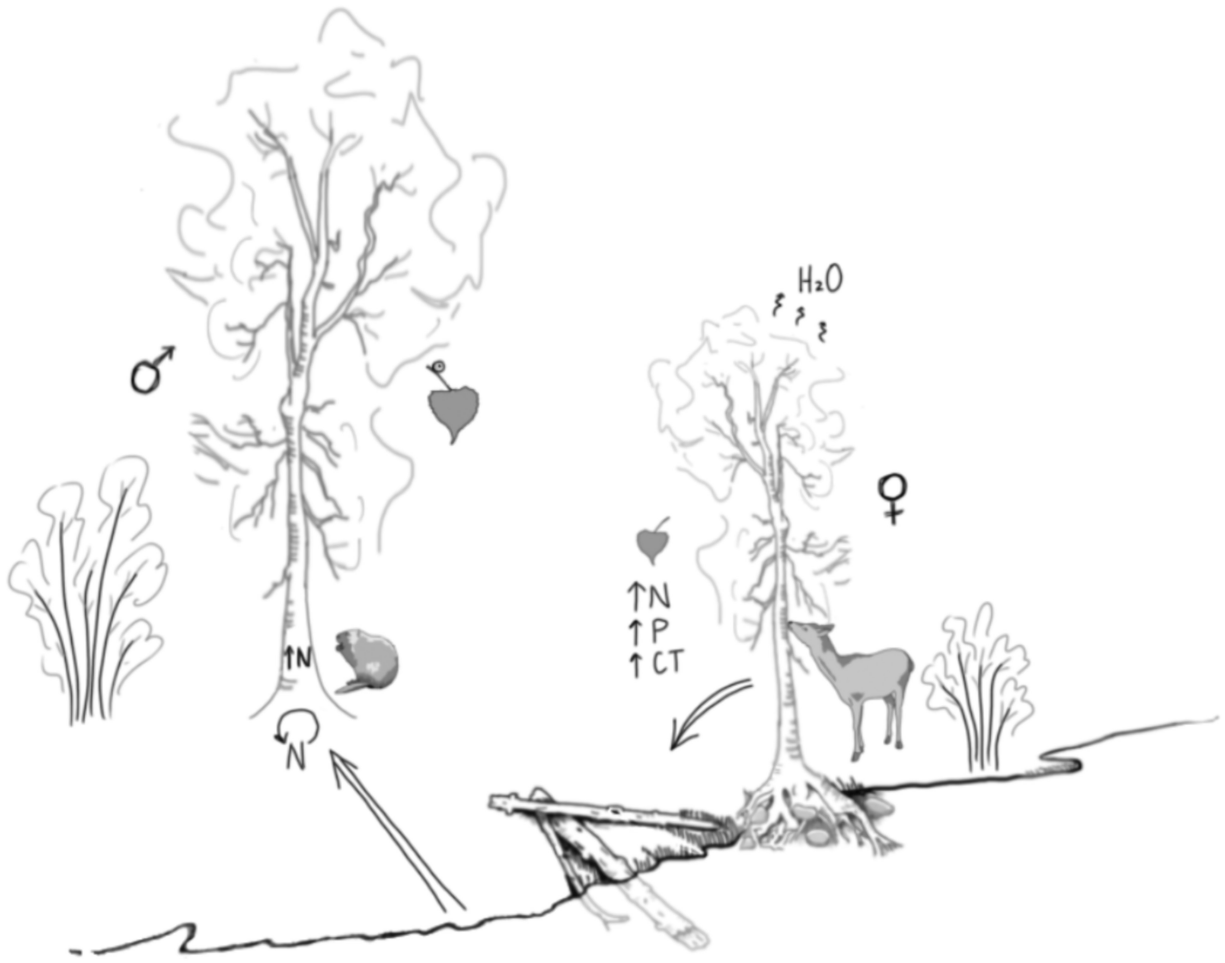
Although there are exceptions to these patterns, our literature review reveals that male riparian trees and shrubs (at left) tend to colonize further away from stream edges, tend to be larger, have larger leaves, more galling insects, higher bark nitrogen, and faster rates of nutrient cycling and potentially selection by beaver. In contrast, female riparian trees and shrubs (at right) tend to colonize closer to stream edges, have higher rates of root growth and arbuscular mycorrhizal colonization, higher rates of mortality and drought stress leading to increased woody recruitment, tend to have both higher leaf‐level nutrients and leaf defense compounds, higher rates of ungulate herbivory, and higher rates of evapotranspiration/water use.

Globally, trees take up and transpire half of the precipitation that falls on the land surface each year (Jackson et al., 2000), and hence even slight variation in water use dynamics can have large ecohydrological implications. Sex‐based dimorphism in water use of riparian trees may influence overall stand‐level transpiration, as well as increase net ecosystem productivity and reduce adjacent stream flows (Figure 2) (Hultine et al., 2007b). Such effects are particularly likely when accompanied by skewed sex ratios (as presented earlier). For example, for streamside female *Acer negundo* (box elder, Sapindaceae), sap flux can be up to 76% higher throughout the entire growing season, leading to overall two times higher whole canopy evapotranspiration compared with males (Hultine et al., 2007a). For *Populus fremontii* (Fremont cottonwood, Salicaceae), sap flux can be 25% higher in females (Hultine et al., 2007b), leading to higher stand‐level transpiration. In both of the above cases, substantial physiological differences are accompanied by skewed sex ratios (Munné‐Bosch, 2015), potentially multiplying the effects at the stand level. Although there are few studies comparing stand‐level water use between male and female individuals for dioecious species, many studies show patterns of skewed sex ratios. Increased transpiration and growth for females might lead to a competitive advantage over males. More generally, in *Salix artica* (arctic willow, Salicaceae), female‐biased sex ratios are increased in more mesic habitats, with males favored in more xeric areas (Dawson & Bliss, 1989). Particularly relevant to riparian ecosystems, females of both *Acer negundo* (boxelder maple, Sapindaceae) and *Salix sitchensis* (Sitka willow, Salicaceae) have been found to grow closer to streams than males (Hultine et al., 2007b; LeRoy, Ramstack Hobbs, et al., 2020). Sex‐based differences in dioecious riparian trees could directly influence ecosystem evapotranspiration, and potentially stream discharge and groundwater recharge (Hultine et al., 2007a, 2007b).

### 7.5 Decomposition

Decomposition is a key ecosystem process that mineralizes organic material and cycles nutrients into available forms. Decomposition in riparian ecosystems (both terrestrial and aquatic) is strongly influenced by litter quality and decomposition rates differ among plant species (Webster & Benfield, 1986), across the phylogenetic tree of life (LeRoy, Hipp, et al., 2020), among genotypes within a species (Jackrel et al., 2016; LeRoy et al., 2006, 2007; Schweitzer et al., 2005), and between plant sexes (LeRoy, Ramstack Hobbs, et al., 2020). Differences in litter quality that lead to increases in decomposition rates include increased leaf litter N (LeRoy, Ramstack Hobbs, et al., 2020), and P, and decreased litter C, lignin, and condensed tannins (LeRoy et al., 2007; Schweitzer et al., 2005; Schweitzer, Bailey, et al., 2008). Leaf‐associated detrital communities can be affected by both litter quality and leaf mass loss, and both aquatic fungi (hyphomycetes; LeRoy et al., 2007) and aquatic macroinvertebrates differentially establish on the leaf litter of genotypes within species of *Populus* (cottonwood, Salicaceae; Whitham et al., 2006). In one study, male *Salix sitchensis* (Sitka willow, Salicaceae) leaves had higher N, lower C:N, and decomposed more quickly (LeRoy, Ramstack Hobbs, et al., 2020).

### 7.6 Adjacent aquatic ecosystem function

Riparian forests are inextricably linked to adjacent stream and river ecosystems (as are lacustrine forests with lakes, and estuarine systems with brackish waters). There are many ways in which riparian trees influence aquatic ecosystem processes and communities and vice versa. Based on the diverse influences of plant sex described above, it is possible that male and female riparian trees may influence adjacent aquatic systems differentially, though there is a paucity of research on this topic (but see LeRoy, Ramstack Hobbs, et al., 2020). Due to differences in plant biomass and architecture, male and female trees may differentially influence stream shading and thus alter stream water temperatures, in‐stream primary production of algae and macrophytes, allochthonous organic matter inputs (wood, leaves, flowers), organic matter decomposition rates, in‐stream nutrient spiraling, channel erosion, and channel dynamics. We will briefly review the literature describing the effects of riparian forests on streams and suggest that examining plant sex‐specific influences would be a productive area of future research.

Riparian trees provide shade to stream ecosystems during leaf out periods. Shade and evaporative cooling in riparian forests can decrease stream water temperatures (Roon et al., 2021) and reduce algal growth in the water column and on the stream bottom (DeNicola et al., 1992; Feminella et al., 1989; Kiffney et al., 2004). Riparian plant shading can also provide habitat and cover for fish and other aquatic vertebrates (Crook & Robertson, 1999; Everett & Ruiz, 1993; Sheldon & Walker, 1998). Riparian trees can help to decrease in‐stream nutrient concentrations through uptake (Fellows et al., 2006; Osborne & Kovacic, 1993; Ramião et al., 2020; Sabater et al., 2000; Schoonover & Lockaby, 2006), decrease overland flows which can reduce in‐stream sediment loads (Wahl et al., 1997), and lower fecal coliform concentrations (Schoonover & Lockaby, 2006). Taken together, riparian forests contribute to improving water quality (Vincent et al., 2016) which has considerable economic value (Piaggio & Siikamäki, 2021; Wang et al., 2017). Riparian shading, when provided by dioecious riparian plants, is likely sex‐differential because although the influence of plant sex on shading has not been directly studied, plant biomass, height, leaf area, architecture, and colonization location can all vary by plant sex (see previous sections). Overall, male plants tend to grow faster and larger (Cornelissen & Stiling, 2005); however, several Salicaceae species dominant in riparian areas exhibit opposite or more neutral tendencies (see Section 3). Finally, female riparian trees are often found closer to stream edges (Hultine et al., 2007a, 2007b; LeRoy, Ramstack Hobbs, et al., 2020), potentially resulting in female trees playing an outsized role in providing shade and other inputs to adjacent aquatic ecosystems.

The diversity (both species‐level and genetic diversity) of riparian forests can influence the quantity, quality, and timing of organic matter inputs (Abelho & Descals, 2019; LeRoy, 2019; LeRoy et al., 2007; Webster & Benfield, 1986). Although there is a paucity of published evidence, it is possible that riparian trees differ by sex in terms of their branch and canopy mortality, susceptibility to beaver (Bailey, Schweitzer, et al., 2004), and contributions of organic matter through adaptations like cladoptosis (DeBell, 1990), all of which would provide differential inputs of organic matter to streams. In addition, inputs of reproductive tissues to detrital pools may differ based on sex. For example, for *Salix sitchensis* (Sitka willow, Salicaceae), female catkins are much larger than male catkins (Fisher, 1928) and female catkin inputs to streams provide variation in the timing, chemistry, and morphology of organic matter inputs compared to leaves (Garthwaite et al., 2021). Variation in leaf litter quality, quantity, and timing can influence in‐stream decomposition rates, microbial communities colonizing leaf litter, and leaf‐shredding invertebrate communities (Graça, 2001; Webster & Benfield, 1986). Organic matter inputs to streams can drive detrital food webs, provide food resources to higher trophic levels like amphibians, crayfish, and fish, and provide sources of dissolved organic matter to downstream reaches. For example, tannins leached from organic matter inputs provided by riparian forests are a key influence on aquatic ecosystem character (Meyer et al., 1987). Inputs of tannins from leaves and woody debris are likely to be influenced by plant sex (Elmqvist et al., 1991) and tannins in aquatic systems can be both potent antimicrobial agents (Scalbert, 1991; Schweitzer, Madritch, et al., 2008) and limit light availability. The potential influences of plant sex on aquatic ecosystem functions described above can also influence aquatic community members, from the smallest bacteria to largest mammalian predators.

## 8 AQUATIC COMMUNITIES

### 8.1 Aquatic microbial communities

Aquatic microbial communities are heavily reliant on organic matter inputs from riparian plants as a carbon source and respond to the chemical and structural properties of these inputs (Gessner & Chauvet, 1994). Microbial communities on plant litter have been shown to differ by genotype and hybrid type for *Populus fremontii* (Fremont cottonwood, Salicaceae) *Populus angustifolia*, (narrowleaf cottonwood), and their F_1_ and backcross hybrids, all of which are dioecious (LeRoy et al., 2007; Marks et al., 2009). These results show the influence of plant genetics on aquatic microbial communities and suggest that plant sex may also be a factor that can structure microbial communities on leaf litter. Although not addressed directly, (LeRoy, Ramstack Hobbs, et al., 2020) found that male and female *Salix sitchensis* (Sitka willows, Salicaceae) differed in terms of phytochemistry and decomposition rate, suggesting possible microbial colonization differences. As microbial communities are affected by the chemical constituents of their substrates, they can also alter the chemical and nutritional properties of leaf litter, conditioning it and making it more nutritious for larger organisms such as shredding aquatic invertebrates (Cornut et al., 2015). Additionally, there are complex interactions between both terrestrial endophytes and aquatic microbes on leaf litter (Hayer et al., 2022) as well as interactions between aquatic microbes and periphytic algae (Halvorson et al., 2019), and both types of interactions with the plant sex of leaf litter warrant further investigation.

### 8.2 Algae and photosynthetic organisms

Algae and photosynthetic organisms in streams provide the base of the green food web and are limited by light availability in streams. Riparian plant canopy cover, species composition, architecture, and life form (evergreen vs. deciduous, shrub vs. tree) can all strongly influence primary production in streams. Shading suppresses algal and periphyton growth (Abe et al., 2003; Mallory & Richardson, 2005), chlorophyll‐a concentrations (Kiffney et al., 2004; Quinn et al., 1997; Roberts et al., 2004), and alters algal community structure (DeNicola et al., 1992; Feminella et al., 1989; Kiffney et al., 2004). For most aquatic macroinvertebrates reliant on algae, shading decreases density, total biomass, and community diversity (Kelly et al., 2003; Quinn et al., 1997; Sturt et al., 2011); however, leaf‐shredding invertebrates (shredders) rely on detrital inputs (Graça, 2001) and leaf piercing invertebrates rely on aquatic plants.

There are several dioecious species of aquatic plants in streams, rivers, and lakes. Most perennial aquatic plants are capable of reproducing both sexually and asexually (Eckert et al., 2016). The paucity of research on dioecious aquatic plants makes it impossible to draw the same connections as we have for terrestrial plants; however, there are a few notable observations. Female *Vallisneria spinulosa* (eelgrass, Hydrocharitaceae) were found to be larger than males, more reproductively limited by low light conditions, and potentially more reproductively influenced by carbon limitation, suggesting a greater capacity for varying resource allocation to growth and reproduction based on environmental conditions (Li et al., 2019). In *Scapania undulata* (aquatic liverwort, Scapaniaceae), females were more resource‐limited, similar to what has been observed for many terrestrial dioecious plants, and sex ratios were male‐biased, perhaps to account for sperm dilution in water (Holá et al., 2014). Many key invasive aquatic plants of the family Hydrocharitaceae are dioecious, including *Elodea canadensis*, *Elodea nuttallii*, *Egeria densa*, *Hydrilla verticillata*, and *Lagorosiphon major*, and it is common to observe a colonizing population consisting of only one sex, reproducing clonally (Eckert et al., 2016; Riis et al., 2010; Sculthorpe, 1967).

### 8.3 Aquatic invertebrates

Several types of aquatic macroinvertebrates may be influenced by the plant sex of either aquatic or riparian plants: piercing herbivores attack aquatic plants, and shredders feed on organic material from mainly riparian plants. Shredders, like the microbial communities discussed above, are influenced by leaf litter inputs (Cummins et al., 1989; Graça, 2001; Motomori et al., 2001). Aquatic insect communities respond to genetic differences in chemical composition of leaf litter, such as tannin concentrations (Whitham et al., 2006) and C:N, which can be significantly higher in female leaf litter (LeRoy, Ramstack Hobbs, et al., 2020). Shading also influences aquatic insect communities; however, for these and other non‐photosynthetic organisms, the effects of shade are less direct and more complicated. One study compared aquatic invertebrates at three differently shaded reaches of a mostly forested coastal British Columbia stream and found increased total invertebrate biomass and community diversity with more shading (Kelly et al., 2003). Another study comparing artificial channels fed from a pastoral New Zealand stream with different shading treatments found shading negatively influenced invertebrate densities and diversity (Quinn et al., 1997). It is likely that the influence of shading differs for aquatic invertebrates in different feeding guilds, benefiting shredders indirectly because they are reliant on organic matter inputs, and negatively influencing herbivores through reductions in algal biomass.

### 8.4 Aquatic vertebrates

For aquatic vertebrates, shading can be important for regulating water temperature, a key factor influencing growth and metabolism. Water temperatures are crucial for many amphibian species who have low critical thermal maxima (Bury, 2008). However, in some cases, excessive shading can be detrimental to tadpole growth (Kiffney et al., 2004). This has practical consequences, as amphibian activity is an indicator used to determine riparian buffer widths (Perkins & Hunter, 2006). Some fish species, such as *Cottus gulosus* (riffle sculpin) and *Salmo trutta* (brown trout), also rely on shading to keep water temperatures below critical values, and generally more shading results in both optimal growth ranges (Baltz & Moyle, 1981; Broadmeadow et al., 2011) and increased ability of fish to evade predators (Helfman, 1981). Other species such as *Sicyopterus japonicus* (grazing goby) prefer unshaded reaches (Abe et al., 2003). For crayfish, shading influences nutrition, with less shade leading to greater consumption of algae and macrophytes, a diet which in laboratory experiments led to faster growth rates (Giling et al., 2009). Within the detrital portion of the crayfish diet, leaves with high N and low C:N are preferentially consumed (Fidalgo et al., 2013). This demonstrates a sensitivity to the chemical composition of vegetative inputs to streams, a sensitivity that also manifests in small crustacean populations in high tannin blackwater streams where calcium availability is limited (Duncan & Fernandes, 2010; Ribeiro & Darwich, 1993). One special freshwater obligate vertebrate, the American beaver (*Castor canadensis*) is considered an ecosystem engineer and can both be influenced by riparian forest conditions, but also alter them.

### 8.5 Beaver as ecosystem engineers

Beavers are large aquatic vertebrates that play a critical role as ecosystem engineers in riparian systems. The dams they build, similar to the roots of riparian trees, can trap sediments and alter hydrodynamics, nutrient flow, and microclimate (Corenblit et al., 2011), and thus influence riparian flora and fauna (Durben et al., 2021). Beavers can affect the composition and succession of riparian forests (Johnston & Naiman, 1990). For example, in cottonwood forests, beaver influence stand‐level genotype ratios (Bailey, Schweitzer, et al., 2004) and differentially select trees at both the species scale (Nolet et al., 1994) and among hybrids of *Populus* (Bailey, Schweitzer, et al., 2004) based on differences in leaf and bark chemistry, trunk size, and establishment location. It is quite possible that beaver also differentially select dioecious trees by sex, but evidence is lacking. Key to understanding this could again be condensed tannin (CT) concentrations, since beavers preferentially select cottonwoods with low CT concentrations (Bailey, Schweitzer, et al., 2004). Whether or not beaver select for specific sexes of riparian trees, beaver influences on riparian forest structure and stream geomorphology are undisputed (Rosell et al., 2005), especially through increased recruitment of large woody debris into streams.

## 9 PHYSICAL STRUCTURE OF RIVERINE SYSTEMS

### 9.1 Large woody debris

The geomorphology of rivers can, under the right conditions, be significantly shaped by riparian trees (Gurnell et al., 2012). Riparian plants both influence and are influenced by fluvial dynamics (Gurnell, 2014) and large woody debris is integral for both processes. Fluvial disturbance is an important driver of large woody debris inputs to streams as flooding and erosion uproots trees along the banks. Once in the channel, this dead wood alters flow, promoting channel avulsion (Collins et al., 2012), and driving the formation of complex, multi‐channel streams (Swanson et al., 2021). These changes influence aquatic communities, algal growth (Sabater et al., 1998), and macroinvertebrate populations (Anderson et al., 1978, 1984; Thompson et al., 2018). In addition, the pools formed by large woody debris create particularly good fish habitat (Fausch & Northcote, 1992), and large woody debris can be added to streams during the construction of beaver dams, contributing to ecosystem engineering and carbon storage (Johnston, 2014; Laurel & Wohl, 2019; Wohl, 2013). While it is poorly understood how plant architecture, cladoptosis, or other factors that influence woody debris character or quantity might differ with plant sex, spatial segregation of the sexes with respect to distance from the streambank (as described above) could point to female trees contributing more to large woody debris recruitment to streams.

### 9.2 Geomorphology and channel dynamics

The flow alterations caused by large woody debris also promote sediment deposition and seedling recruitment (Gregory, 2000; Gregory & Ashkenas, 1990). Pioneering plants can trap and stabilize sediment (Corenblit et al., 2011; Gurnell et al., 2012), driving pioneer landform development (Collins et al., 2012). While the roots of pioneering trees aggrade sediments for new landforms, the roots of established trees along riverbanks alter bank erosion regimes, and thus shape bank profiles (Davis & Gregory, 1994; Grabowski & Gurnell, 2016; Rutherfurd & Grove, 2004). The root structures of riparian plants are recognized as key to stabilizing riverbanks and increasing sheer strength (Andreoli et al., 2020). For example, female trees tend to grow closer to riverbanks and, in *Populus cathayana* (Manchurian poplar, Salicaceae) females exhibited greater root mass and root allocation, particularly when neighboring roots were also female (Dong et al., 2017). Given the female‐biased sex ratios found near riverbanks, female roots growing near female root neighbors are more likely to be found in this crucial streamside environment. In addition, female roots growing in proximity with other female roots were linked with increased root diameters but decreased specific root length and branching intensity (Dong et al., 2017). These patterns of root growth for female roots growing near other female roots suggest female trees may be producing more of the coarse roots that are crucial for stabilizing banks and shaping channel dynamics. In addition, since riparian species can have extensive clonal spread, this could cause biased ramet sex ratios.

### 9.3 Baseflow and surface runoff

Beyond influencing how water flows within streams, riparian plants can influence both the baseflow to nearby streams (through evapotranspiration losses, but also shading) and the infiltration of water into the soil beneath them (through organic matter inputs, root growth, and hydraulic lift; Lange et al., 2009). Key dioecious phreatophytic plants including willows, cottonwoods, and alders transpire large quantities of water, reducing adjacent stream baseflows (Hultine et al., 2007b). In addition, given the documented intersexual differences in water use and spatial segregation of the sex of trees adjacent to streams, there is additional potential for plant sex to influence baseflows. Greater water demand by females can lead to female‐biased sex ratios near streambanks, and thus more pronounced influences on baseflow, particularly in mesic environments (Hultine et al., 2007a, 2007b). Increased transpiration that decreases baseflow can, in turn, drive an increase in infiltration (Berland et al., 2017), thereby increasing the water storage capacity of soils (Lange et al., 2009). Increased infiltration rates under trees have also been attributed to roots and the channels they form (Kazemi Zadeh & Sepaskhah, 2016). This has been specifically observed for the dioecious *Ziziphus spina‐christi* (Christ's thorn tree; Al‐Maktoumi et al., 2020) the dioecious *Olea europaea* (common olive, Oleaceae; Vanderlinden et al., 1998). Documented intersexual differences in transpiration, and in some cases, root allocation and growth, suggest promise for plant sex influencing infiltration. However, broader intersexual differences in infiltration rates or hydraulic conductivity of surrounding soils have yet to be studied. Knowing how plant sex influences evapotranspiration, infiltration, and baseflow would help us understand how riparian ecosystems interact with intermittency and drought, which are projected to be increasingly common as the climate changes.

## 10 IMPLICATIONS

### 10.1 Climate change influences

Climate change influences on stream and riparian ecosystems are predicted to be variable across landscapes, but include changes to drought regimes, water availability, temperature, CO_2_ concentrations, and result in increased stream intermittency and plant range shifts (Pörtner et al., 2022). Temperature and CO_2_ concentrations have been linked with concrete intersexual influences on dioecious plants that will likely shape riparian communities going forward, both changing sex ratios (Munné‐Bosch, 2015), and physiological responses. For example, elevated temperatures can drive 70–100% increases in flavonoid and tannin content in female *Salix myrsinfolia* (dark‐leaved willow, Salicaceae; Randriamanana et al., 2014). This has the potential to amplify the myriad of tannin‐related interactions described in previous sections. Linked with increases in temperature are increases in atmospheric CO_2_ concentrations, which in combination caused a doubling of net carbon assimilation rates in male *Salix arctica* (arctic willow, Salicaceae) compared with females, as seen for significant temperature*sex effects in Jones et al. (1999). As another example, in *Populus cathyana* (Manchurian poplar, Salicaceae), males benefitted more from CO_2_ enrichment than females (Zhao et al., 2021), and elevated CO_2_ led to more pronounced increases in male *Populus tremuloides* (trembling aspen, Salicaceae) photosynthetic rates and total biomass, compared to females (Wang & Curtis, 2001). Shifting climates have the capacity to alter many leaf‐level plant traits, with consequences for nutrient cycling and decomposition both on the forest floor, and in adjacent aquatic ecosystems (Jeplawy et al., 2021).

### 10.2 Evolutionary consequences

Examples of climate changes that will strongly influence riparian ecosystems are increasing droughts, floods, and stream intermittency. As we have outlined, female plants tend to be more susceptible to drought, with the potential for shifting sex ratios and thus changes in riparian ecosystem structure. One example is on the Yakima River in eastern Washington where decades of regulated flow regimes have negatively influenced habitat quality and have been correlated with skewed sex ratios favoring male *Populus trichocarpa* (black cottonwood, Salicaceae; Braatne et al., 2007). Males are often more tolerant of poorer quality habitat, and this combined with relatively better performance under elevated temperatures and CO_2_ conditions could produce increasingly male‐biased sex ratios for certain riparian species. Similar patterns have been observed in the herbaceous *Valeriana edulis* (tobacco root, Caprifoliaceae), for which male frequency has been increasing by 1.28% per decade, a rate that is outpaced by the changing conditions brought on by climate change (Petry, 2016). As such, climate change, acting as an agent of selection, could lead to excessively skewed sex ratios, hamper reproductive success, and reduce resilience to desertification (Jiang et al., 2016). Dioecious riparian plants responding to climate changes and selection by herbivores, combined with their ability for shaping the physical structure of riparian systems, highlight the possibility that key dioecious riparian plants might be involved in driving successionary change (Corenblit et al., 2009) and potentially contribute to the processes of evolutionary geomorphology—feedbacks among geomorphologic, ecological, and evolutionary processes through organism‐driven landform modifications (Steiger & Corenblit, 2012).

### 10.3 Conservation and restoration

Riparian and riverine systems are, in many cases, dominated by human influences and broadly in need of restoration. Restoration strategies over the past 50 years have given increasing importance to issues of genetic variation from source populations for restoration propagules, but only recently have begun addressing other key life history strategies during restoration (Palmquist et al., 2021). Specifically, through broadscale surveys of genetic variation along rivers, recent research has found that mating systems, clonality, and seed dispersal methods are all related to genetic structure in riparian habitats (Palmquist et al., 2021). Given the important roles dioecious plants play in linked riparian and adjacent stream ecosystems, plant sex should be a major consideration in designing restoration efforts as well as planning landscape‐scale sex ratios for climate resilience in the future. For plants that reproduce clonally, it may be possible to determine the sex of collected vegetative material and plant landscapes with natural ratios of males to females, or with ratios that may better resist future climate changes. Studies of plant sex should follow the growing body of research on genetic variation within species and assisted migration of riparian plants for restoration purposes (Keith et al., 2023).

## 11 CONCLUSIONS

Although there is a substantial body of research on the influences of dioecy on plant morphological, chemical, and physiological traits, there are several gaps in the literature on the influences on terrestrial and aquatic communities and ecosystem processes that were revealed by this review. Additionally, this review raises questions about differences in sex‐based traits between shrubs and trees, between temperate zones and the tropics (very few studies of tropical dioecious plants exist, but we attempted to review them here), and the determinants of sex bias at landscape scales. We do not yet understand the mechanisms for biased sex ratios in most species and various factors (differential germination, differential mortality, differential clonal reproduction, differential resistance to herbivores and pathogens, and environmental determinism of sex expression) could contribute to biased sex ratios on the landscape, so more detailed studies of these mechanisms are needed. In particular, why might female riparian plants be more dominant in streamside habitats? The mechanisms for this sex‐ratio bias are an important area of future research.

Overall, our literature review reveals several potential influences of plant dioecy on riparian and aquatic communities and ecosystems. Specifically, riparian plant dioecy influences: (1) plant‐level traits: male plants are generally larger, have larger leaves, and female plants have higher leaf‐level nutrients and leaf defense compounds; (2) terrestrial community members: male plants support more galling insects and female plants support higher levels of arbuscular mycorrhizal colonization and ungulate herbivores; (3) riparian ecosystem processes: male plants have faster rates of terrestrial nutrient cycling and leaf litter decomposition and female plants have higher rates of evapotranspiration; (4) aquatic community members: the female sex ratio bias increases toward stream edges, creating shade, altering structural habitat, and providing more leaf litter and woody debris inputs to streams than males, which serve as energy resources for stream communities; (5) the physical structure of riverine systems: female plants have higher rates of root growth and mortality (leading to woody recruitment), influencing bank erosion and channel dynamics, as well as higher rates of stand‐level water use which can influence stream baseflows. Overall, these broad influences of dioecy have implications for climate changes in riparian habitats, the evolution and range shifts of riparian species, and the methods used for conserving and restoring riparian systems.

## AUTHOR CONTRIBUTIONS

**River Scheuerell:** Conceptualization (equal); data curation (equal); methodology (equal); writing – original draft (lead); writing – review and editing (supporting). **Carri J. LeRoy:** Conceptualization (equal); data curation (equal); methodology (equal); project administration (lead); supervision (lead); visualization (lead); writing – original draft (supporting); writing – review and editing (lead).

## Data Availability

Data sharing not applicable to this article as no datasets were generated or analysed during the current study.
